# PERK-Dependent Activation of the JAK2/STAT3 Pathway Contributes to High Glucose-Induced Extracellular Matrix Deposition in Renal Tubular Epithelial Cells

**DOI:** 10.1155/2021/8475868

**Published:** 2021-07-19

**Authors:** Yan Bao, Wei Liang, Yingchun Ye, Bo Yi

**Affiliations:** ^1^Department of Endocrinology, Renmin Hospital of Wuhan University, Wuhan 430060, Hubei, China; ^2^Department of Nephrology, Renmin Hospital of Wuhan University, Wuhan 430060, Hubei, China

## Abstract

**Background:**

Although the deposition of extracellular matrix (ECM) is critical leading to tubular damage in diabetic kidney disease (DKD), the mechanism still remains unclear. The purpose of this study was to demonstrate a role for protein kinase R-like endoplasmic reticulum kinase (PERK) (a protein located in the endoplasmic reticulum membrane) in this pathologic process.

**Methods:**

NRK-52E cells were grown in the media containing different concentrations of glucose or thapsigargin for different durations. Cells were subsequently incubated with or without AG490, a selective inhibitor of Janus kinase 2 (JAK2) or GSK2606414 (a selective PERK inhibitor). We evaluated the production of TGF-*β*1, fibronectin, and collagen I proteins by ELISA. The levels of 78 kD-glucose-regulated protein (GRP78) and PERK, as well as the phosphorylation statues of PERK and JAK2/signal transducer and activator of transcription (STAT3), were determined by western blotting analysis.

**Results:**

We showed that the increased phosphorylation of JAK2 and STAT3 was accompanied by overexpression of TGF-*β*1 and ECM deposition in high glucose medium. Disruption of the JAK2/STAT3 pathway with AG490 significantly prevents the high glucose-induced increase in TGF-*β*1, fibronectin, and collagen I. High glucose induced the overproduction of GRP78 and phosphorylation of PERK, which indicated that endoplasmic reticulum stress (ERS) was triggered in NRK-52E cells cultured under high glucose condition. Inhibition of PERK phosphorylation with GSK2606414, however, blocked the effect of JAK2/STAT3 on the production of TGF-*β*1 and ECM components in NRK-52E cells.

**Conclusion:**

Our data indicated that the ECM accumulation induced by high glucose arouse via the PERK-dependent JAK2/STAT3-signaling pathway in renal tubular epithelial cells.

## 1. Introduction

Diabetic kidney disease (DKD) is a serious chronic microvascular complication of diabetes and is the leading cause of end-stage renal disease (ESRD) [1]. Although DKD is characterized by glomerular and tubular epithelial hypertrophy and the accumulation of extracellular matrix (ECM), the severity of tubulointerstitial lesions is more closely related to proteinuria and a progressive diminution in renal function. Some diabetic patients still develop progressive renal damage even though their metabolic abnormalities are well controlled. One of the mechanisms underlying such damage is hypothesized to be the initiation of tubular epithelial cell injury by high glucose concentration, accompanied by the overproduction of TGF-*β*1 and ECM components, such as collagen and fibronectin (FN) [2]. Therefore, preventing the accumulation of ECM in the tubulointerstitium is critical to improving the accelerated progression of DKD.

The most potent simulator of ECM accumulation is transforming growth factor-*β*1 (TGF-*β*1), a cardinal profibrotic member of the TGF-*β* superfamily [3]. This is considered to be an essential factor in the development of DKD in diabetic animals and patients [4]. Wang et al. [5] have revealed that high glucose can stimulate TGF-*β*1 in rat glomerular mesangial cells through the Janus kinase 2 (JAK)/signal transducer and activator of transcription (STAT) pathway. Experimental evidence suggests that inhibition of JAK/STAT (in particular, JAK2/STAT3) may suppress renal fibrosis and thereby protect renal function [6]. The JAK/STAT signaling pathway, activated by its phosphorylation, is closely related to the production of TGF-*β*1, FN, and collagen [7]. Fibronectin and collagen are the primary components of the ECM. The FN protein is composed of two peptide chains that attach cells to a variety of ECM components [8]. However, research on the role of JAK/STAT signaling in renal damage has been inconsistent.

Endoplasmic reticulum stress (ERS) has been widely indicated to play an essential role in kidney injury [9, 10]. The endoplasmic reticulum (ER) is the processing center where newly synthesized proteins are correctly folded and assembled. Adverse stimuli such as hyperglycemia, oxidative stress, and hypoxia can lead to the accumulation of unfolded or misfolded proteins in the ER lumen and ultimately drive ER stress, which is recognized to be an adaptive response [11]. However, when ERS is prolonged and severe, it can lead to a variety of diseases, including DKD and acute kidney injury [12]. Malfunctions of the ER induced by a variety of factors can lead to the unfolded protein response (UPR), resulting in ERS. The UPR is initiated by three transmembrane transducers, one of which is protein kinase R-like endoplasmic reticulum kinase (PERK). The interactions between ERS and STAT signaling are currently receiving increasing attention. Recent evidence suggests that the PERK/JAK1/STAT3 signaling pathway is a driver of neuroinflammation and that this may be relevant to multiple sclerosis [13]. However, it remains unclear as to whether ERS can activate the JAK/STAT pathway in the kidney. In this study, we investigated the relationship between ERS and JAK/STAT and explored their roles in high glucose-induced ECM deposition in renal tubular epithelial cells.

## 2. Materials and Methods

### 2.1. Experimental Materials

Anti-GRP78, PERK, phosphor-PERK, JAK2, and STAT3 antibodies were purchased from Santa Cruz Biotechnology (Santa Cruz, CA, USA). We purchased thapsigargin from Sigma (USA) and GSK2606414 and AG490 from Selleck Chemicals (USA). TGF-*β*1 and FN ELISA kits were bought from Boster (Wuhan, China), a collagen I ELISA kit was from BlueGene Biotech. (Shanghai, China), and a cell counting kit (CCK-8) was bought from Dojindo (Kumamoto, Japan). Rat renal proximal tubule epithelial cells (NRK-52E) were obtained from the American Type Culture Collection.

### 2.2. Cell Culture

NRK-52E cells were cultured in Dulbecco Modified Eagle's Medium (DMEM) supplemented with 10% of fetal bovine serum, glucose (5.6 mM), 100 mg/mL of streptomycin, and 100 units/ml of penicillin at 37°C, in a 5% CO_2_ atmosphere. Cells were seeded at a density of 1 × 10^5^ cells/well in 6-well dishes and were transferred to the serum-free medium for 24 hours for further experiments.

### 2.3. Enzyme-Linked Immunosorbent Assay

TGF-*β*1, FN, and collagen I were examined using ELISA kits according to the manufacturers' instructions. Each experiment was repeated three times.

### 2.4. Western Blot Analysis

These methods were performed according to our previously published study [14]. After completing experiments, the medium was removed and the cells were washed twice with ice-cold PBS. Cells were then lysed with RIPA lysis buffer (Beyotime biotechnology, China), containing 1 mmol/L PMSF and quantified using a bicinchoninic acid protein assay kit (Beyotime Biotechnology, China). Cell homogenates were separated by 10% SDS-PAGE and transferred to polyvinylidene fluoride membranes, which were then blocked for 1 h at room temperature with 5% skim milk powder in TBST. The blots were incubated with one of the following primary polyclonal antibodies: GRP78, phosphor-PERK, phosphor-JAK2, or phosphor-STAT3. Horseradish peroxidase-conjugated goat anti-rabbit immunoglobulin G was used as the secondary antibody. After the chemiluminescence reaction, the bands were detected by exposing the blots to the X-ray film. The same membrane was then reused to determine *β*-actin by incubating it with a monoclonal anti-*β*-actin antibody. For quantitative analysis, the bands were detected and evaluated densitometrically with BandScan software (Glyko Inc., Novato, CA, USA) and normalized to the corresponding density of *β*-actin. Every result was issued from three independent experiments.

### 2.5. CCK-8 Assay

The cellular suspensions (100 *μ*L) were added to each well of a 96-well plate. After they adhered, cells were synchronized for 24 h and cultured with different concentrations of glucose. The medium was discarded, and the plate was washed twice with phosphate-buffered saline (PBS). Ten microliters of CCK-8 and 90 *μ*L fresh medium were added to each well of the plate. The cells were subsequently incubated for 4 h at 37°C, and absorbance was measured at 450 nm. Three independent experiments were performed.

### 2.6. Statistical Analysis

We expressed all data as means ± SE. Data were compared among groups using one-way ANOVA followed by Tukey's multiple-comparison test with SPSS statistical software version 21.0 (SPSS Inc., Chicago, USA). A value of *P* < 0.05 was considered statistically significant.

## 3. Results

### 3.1. Overexpression of TGF-*β*1, FN, and Collagen I Induced by High Glucose Is JAK2/STAT3-Dependent in NRK-52E Cells

In this experiment, we cultured NRK-52E cells in different concentrations of glucose (NC group, 5.6 mM; H15 group, 15 mM; H25 group, 25 mM; and H50 group, 50 mM) or mannitol (Mann group, 5.6 mM D-glucose + 19.4 mM D-mannitol) for 12 h, 24 h, or 48 h. The presence of higher levels of glucose augmented the levels of TGF-*β*1 and the components of the ECM, while the expression of the above proteins did not change in the control or mannitol groups (Table 1). Because the concentrations of the aforementioned proteins were highest when cells were cultured in 25 mM glucose for 24 hours, the subsequent experiments were selected on this basis (Table 1).

**Figure 1:**
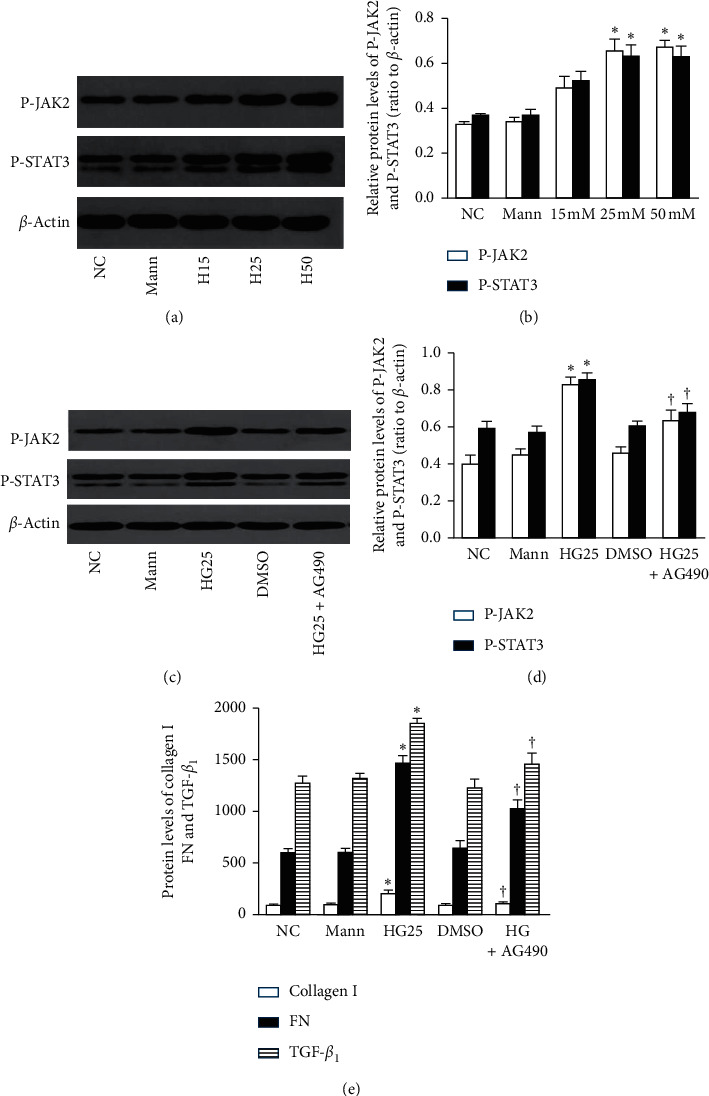
High glucose increases the protein levels of TGF-*β*1, FN, and collagen I through the phosphorylation of the JAK2/STAT3 pathway (*n* = 3). (a, b) High glucose facilitated the phosphorylation of JAK2 and STAT3. (c, d) AG490 (100 *μ*M) inhibited JAK2/STAT3 phosphorylation and (e) reduced the overexpression of TGF-*β*1, FN, and collagen I proteins (^*∗*^*P* < 0.05 compared with the NC group; ^†^*P* < 0.05 compared with the HG25 group).

Our results also showed that high glucose facilitated the phosphorylation of JAK2 and STAT3 (*P* < 0.05, Figures 1(a) and 1(b)). To determine whether the overexpression of TGF-*β*1 and ECM induced by high glucose occurred by virtue of the JAK2/STAT3 pathway, we cocultured cells with AG490 (a selective inhibitor of JAK2) and observed that AG490 (100 *μ*M) suppressed high glucose-induced phosphorylation of JAK2/STAT3 (*P* < 0.05) (Figures 1(c) and 1(d)) and reduced TGF-*β*1, FN, and collagen I protein levels (*P* < 0.05, Figure 1(e)). This indicated that overproduction of TGF-*β*1, FN, and collagen I induced by high glucose was JAK2/STAT3-dependent in NRK-52E cells.

### 3.2. High Glucose Triggers ER Stress

**Figure 2:**
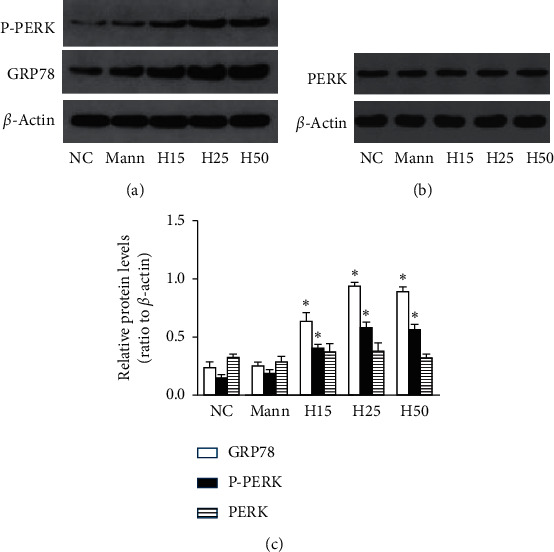
ERS is triggered by high glucose (*n* = 3). (a, c) The expression of GRP78 and phosphorylation of PERK were increased under high glucose conditions, (b, c) while PERK protein did not change (^*∗*^*P* < 0.05 compared with the NC group).

Compared with the control group, although the expression of GRP78 protein and phosphorylation of PERK were enhanced under high glucose conditions (*P* < 0.05, Figure 2), PERK protein did not change. These results are congruent with our previous work, suggesting that ERS was triggered by high glucose [14].

### 3.3. Overexpression of TGF-*β*1 and ECM Deposits during ERS Are JAK2/STAT3-Dependent

**Figure 3:**
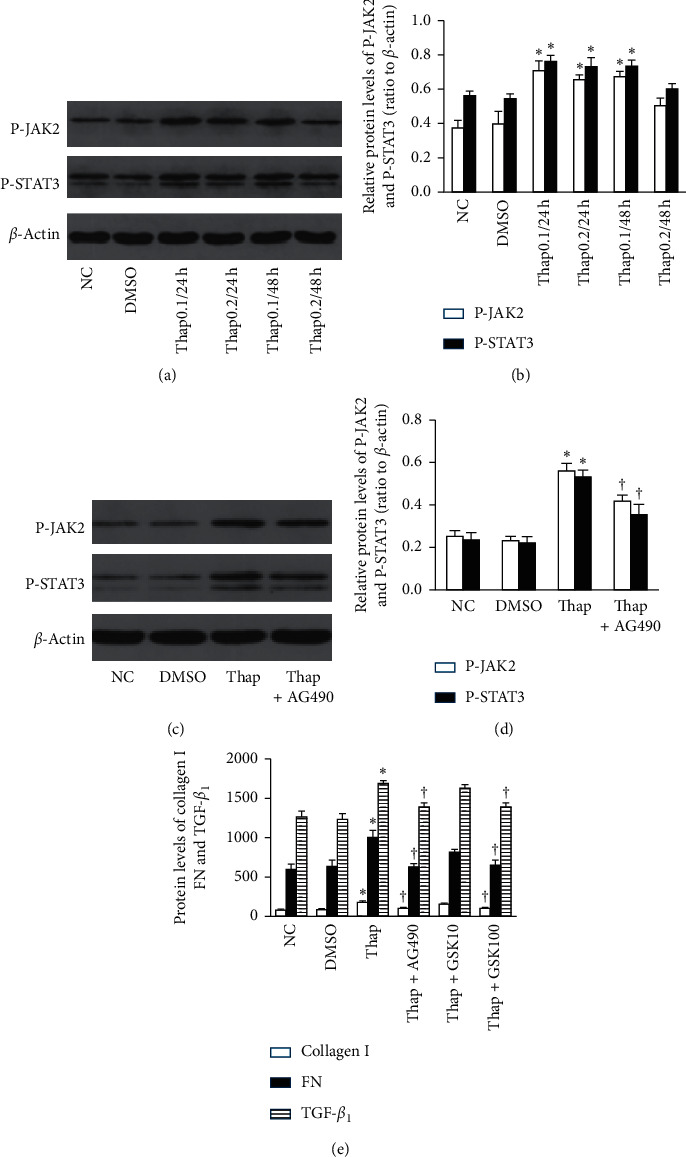
Overexpression of TGF-*β*1 and ECM deposits during ERS are JAK2/STAT3-dependent (*n* = 3). (a, b) Thapsigargin accelerated the phosphorylation of JAK2 and STAT3. (c, d) AG490 (100 *μ*M) blocked the JAK2/STAT3 phosphorylation as well as (e) the overexpression of TGF-*β*1, FN, and collagen I (^*∗*^*P* < 0.05 compared with the NC group; ^†^*P* < 0.05 compared with the thap group).

To investigate the role of the JAK2/STAT3 signaling axis on ERS-induced overproduction of TGF-*β*1 and the components of the ECM, NRK-52E cells were treated with thapsigargin (0.1 *μ*M and 0.2 *μ*M) for 6 h, 24 h, or 48 h. Thapsigargin augmented protein levels for TGF-*β*1, FN, and collagen I (Table 2); when cells were incubated in 0.1 *μ*M thapsigargin for 24 h, the levels of TGF-*β*1 and ECM were higher than in the other groups (Table 2). In addition, thapsigargin accelerated the phosphorylation of JAK2 and STAT3 (*P* < 0.05, Figures 3(a) and 3(b)). We also observed that, for cells cocultured with AG490 (100 *μ*M) and thapsigargin, AG490 inhibited the phosphorylation of JAK2 and STAT3 induced by thapsigargin (*P* < 0.05, Figures 3(c) and 3(d)), as well as the levels of TGF-*β*1 protein and ECM (*P* < 0.05, Figure 3(e)). Collectively, these results indicated that the overproduction of TGF-*β*1 and ECM stimulated during ERS was mediated by the JAK2/STAT3 pathway.

### 3.4. The Activation of JAK2/STAT3 during ERS Is Partially PERK-Dependent

**Figure 4:**
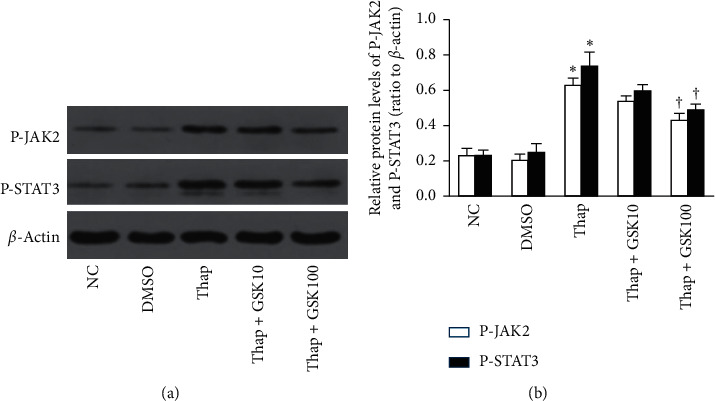
The activation of JAK2/STAT3 during ERS is partially PERK-dependent (*n* = 3). Cells were pretreated with GSK2606414 for 30 minutes and cocultured with thapsigargin. GSK2606414 (100 nM) reduced the phosphorylation of JAK2/STAT3 (^*∗*^*P* < 0.05 compared with the NC group, ^†^*P* < 0.05 compared with the thap group).

To determine the role of PERK in the activation of JAK2/STAT3, we pretreated cells with GSK2606414 (a potent selective inhibitor of PERK) and cocultured them with thapsigargin. GSK2606414 (100 nM) arrested the phosphorylation of JAK2 and STAT3 significantly (*P* < 0.05, Figure 4) accompanied by diminution in TGF-*β*1 protein and ECM components (*P* < 0.05, Figure 3(e)). These data suggested that the PERK branch of the UPR in ERS was important in facilitating the phosphorylation of JAK2 and STAT3.

### 3.5. High Glucose Upregulates the Deposit of ECM via PERK-Dependent JAK2/STAT3 in NRK-52E Cells

**Figure 5:**
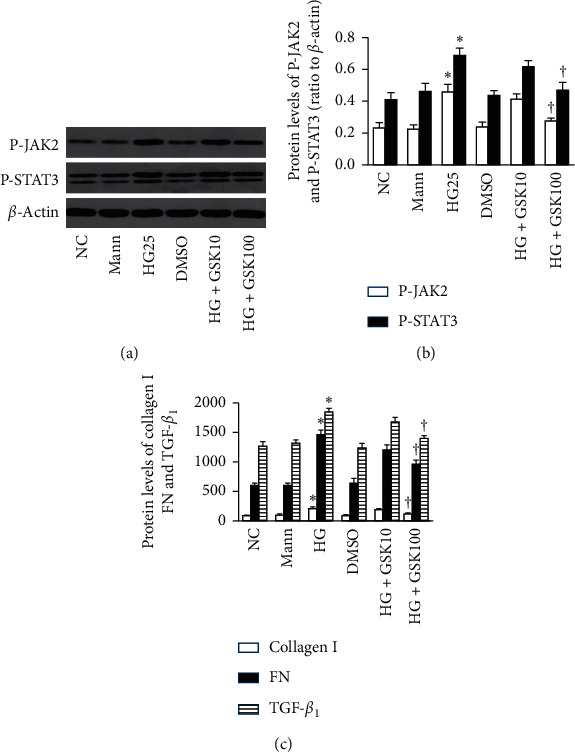
High glucose facilitates the deposit of ECM via the PERK-dependent JAK2/STAT3 pathway in NRK-52E cells (*n* = 3). Cells were pretreated with different concentrations of GSK2606414 (10 nM, 100 nM) for 30 minutes and coincubated with 25 mmol/L glucose for 24 h. (a, b) The higher concentration of GSK2606414 (100 nM) inhibited the phosphorylation of JAK2 and STAT3 induced by high glucose as well as TGF-*β*1, FN, and collagen I proteins (c) (^*∗*^*P* < 0.05 compared with the NC group; ^†^*P* < 0.05 compared with the HG25 group).

NRK-52E cells were pretreated with GSK2606414 (10 nM and 100 nM) for 30 minutes and coincubated with 25 mM glucose. When cells were treated with GSK2606414 under a high glucose condition, the phosphorylation of JAK2/STAT3 was partially blocked (*P* < 0.05, Figures 5(a) and 5(b)) along with TGF-*β*1, FN, and collagen I proteins (*P* < 0.05, Figure 5(c)). Collectively, these results indicated that TGF-*β*1 and ECM accumulation induced by high glucose was partially mediated via the PERK/JAK2/STAT3 pathway.

### 3.6. Cell Viability under Different Concentrations of Glucose

**Figure 6:**
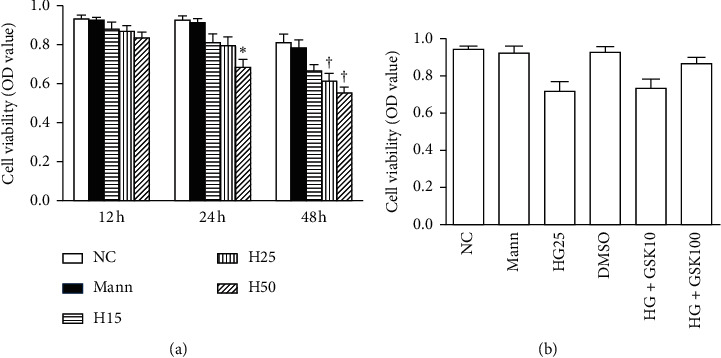
Cell viability in different concentration of glucose (*n* = 3). (a) NRK-52E cells were cultured in the medium with different concentrations of glucose or mannitol for 12 h, 24 h, or 48 h (^*∗*^*P* < 0.05 compared with 5.6 mmol/L group at 24 h; ^†^*P* < 0.05 compared with 5.6 mmol/L group at 48 h). (b) NRK-52E cells were pretreated with GSK2606414 (10 nM and 100 nM) for 30 minutes and coincubated with 25 mmol/L glucose for 24 h. GSK2606414 treatment did not decrease the cell viability.

The NRK-52E cells were cultured in the medium with different concentrations of glucose or mannitol for 12 h, 24 h, or 48 h. Compared with the control group, the results of the CCK-8 assay indicated an attenuation of cellular activity in the H25 group at 48 h and in the H50 group at 24 h and 48 h (*P* < 0.05, Figure 6(a)). We did not observe a reduction in cellular viability in the H25 group at 12 h and 24 h or in the H15 group for different hours. Then, NRK-52E cells were pretreated with GSK2606414 (10 nM and 100 nM) for 30 minutes and coincubated with 25 mmol/L glucose for 24 h. The cell viability was not affected by GSK2606414 (Figure 6(b)).

## 4. Discussion

DKD is a serious complication of diabetes, with a major morphologic feature being the change in ECM. Owing to increased amounts of ECM, basement membranes are thickened, the glomerular mesangial matrix and tubulointerstitial space are expanded, which ultimately led to failure of kidney function [15]. TGF-*β*1 is the central cytokine in many cellular processes, and accumulating evidence reveals that TGF-*β*1 is closely related to renal ECM deposits in DKD [16]. High glucose can upregulate TGF-*β*1 protein in renal tubular epithelium, which then induces ECM accumulation and delays its degradation. The results of our experiment were consistent with those of previous studies, as TGF-*β*1, FN, and collagen I were all overproduced under high glucose conditions.

It is, however, unclear that how high glucose induces the expression of TGF-*β*1 and ECM accumulation. The JAK/STAT pathway is a crucial signal transduction cascade that regulates cellular activation, proliferation, and inflammation, and the activation of the JAK/STAT-signaling pathway leads to the development of fibrosis in the lung and kidney via the induction of TGF-*β*1 and collagens [17, 18]. A STAT3 inhibitor suppressed TGF-*β*1 so as to prevent the occurrence of DKD in STZ-induced rats [19], while it was also reported that JAK2/STAT3 signaling may play a role in the renal fibrosis repair process in mice with UUO (unilateral ureteral obstruction) and that this effect was partially mediated by MMP-2 activation. This article suggested that TGF-*β*1 may not be a molecular target of the JAK/STAT axis [20]. Therefore, to clarify the role of JAK2/STAT3 in the synthesis of TGF-*β*1 and ECM, NRK-52E cells were herein cultured with high glucose and concomitantly incubated with AG490. Our results revealed that AG490 inhibited the phosphorylation of JAK2/STAT3, accompanied by a decrease in TGF-*β*1 protein and ECM components. These data indicated that although the overproduction of TGF-*β*1 and ECM induced by high glucose was JAK2/STAT3-dependent, the underlying mechanism is arcane and requires further elucidation.

ERS has emerged as an important pathophysiologic phenomenon that underlies metabolic diseases, such as diabetes, and GRP78 is a vital molecular indicator of ERS. The most immediate response to ER stress is the homodimerization and transphosphorylation of PERK [21]. Our previous results showed that high glucose elevated the GRP78 protein and PERK phosphorylation. As mannitol exerted no effect on either of these proteins, this suggested us that these effects were not induced by hyperosmosis [14].

Moderate ER stress can maintain cellular homeostasis. However, severe or prolonged ERS ultimately induces deterioration of cellular function and cell death. ERS has been documented as being intimately associated with inflammation and stress signaling networks [22]. There is evidence that the interactions between ERS and STAT singling may be exquisitely intertwined. ERS can trigger STAT3 in the acute-phase response of infection, which may then facilitate the processing and delivery of newly synthesized loads of acute-phase proteins, and thus prevent liver from injury during infection [23]. ERS is known to activate STAT3 and NF-*κ*B [24]. Both PERK and IRE1*α* can activate STAT3, which in turn promotes survival through upregulation of antiapoptotic protein [25]. PERK is specifically activated in response to ER stress in neighboring cells of intestinal stem cells (ISC) through JAK/STAT signaling, which can limit *Drosophila* lifespan by promoting ISC proliferation [26]. Since our study showed that thapsigargin promoted the phosphorylation of JAK2 and STAT3, we subsequently used a potent selective PERK inhibitor (GSK2606414) to pretreat cells. After coculture with GSK2606414, the increased levels of JAK2 and STAT3 induced by thapsigargin declined significantly. These results showed that ERS elevated the phosphorylation of JAK2 and STAT3 partly through the PERK branch of UPR. Additionally, TGF-*β*1, FN, and collagen I proteins induced by thapsigargin were all blocked by GSK2606414. These results revealed that ERS upregulated TGF-*β*1 and ECM proteins, and that these actions were partially mediated through the PERK/JAK2/STAT3 pathway.

However, it is still unclear as to how high levels of glucose induced the accumulation of ECM through ER stress in DKD. Recent studies have revealed that ERS plays a crucial role in renal fibrosis, which can be improved by amelioration of ERS. [27]. It has also been reported that ER stress appears to occupy a critical role in activation of the albuminuria-induced inflammasome. Elimination of ERS might thereby serve as a novel avenue for ameliorating kidney epithelial cell injury induced by albuminuria [28]. Qi et al. [29] demonstrated that 4-PBA exerted a notable renoprotective effect by lowering the expression of p-PERK in STZ-induced diabetic rats. Moreover, P58^IPK^ is a vital component whose function was ascertained by suppressing PERK activation during ERS. Yang et al. [30] also established a protective role for P58^IPK^ against ER stress-mediated diabetic retinopathy. In our study, when NRK-52E cells were cultured for 24 h with high glucose in the presence or absence of GSK2606414, we showed that GSK2606414 treatment suppressed the increase in phosphorylation of JAK2 and STAT3 induced by high glucose. We also showed that this was accompanied by the downregulation of TGF-*β*1 protein and constituents of the ECM.

In conclusion, our findings revealed that high glucose induced an increase in the synthesis of TGF-*β*1 and ECM deposits through the JAK2/STAT3-signaling pathway, and this increase was partially mediated by the PERK branch of the unfolded protein response in ERS. Since we traditionally recognize that TGF-*β*1 and the accumulation of ECM contribute to the deterioration of renal function, our study suggests that inhibiting PERK during ER stress might constitute a potential novel therapy for improving DKD.

## Figures and Tables

**Table 1:** The protein levels of TGF-*β*_1_, FN, and collagen I in different concentrations of glucose at different times.

	TGF-*β*1 (ng/L)		FN (*μ*g/ml)		Coll I (ng/L)	
	Mean ± SD	*P*	Mean ± SD	*P*	Mean ± SD	*P*
NC/12 h	1277.52 ± 126.83	—	578.46 ± 62.22	—	82.47 ± 6.83	—
Mann/12 h	1259.70 ± 154.35	1.00	575.66 ± 113.15	1.00	85.50 ± 8.81	1.00
H15/12 h	1435.74 ± 144.83	0.99	1089.94 ± 171.20^*∗*^	<0.05	123.99 ± 15.18^*∗*^	<0.05
H25/12 h	1912.33 ± 244.32^*∗*^	0.02	1185.45 ± 252.14^*∗*^	<0.05	163.42 ± 10.55^*∗*^	<0.05
H50/12 h	1591.20 ± 200.31	0.76	1042.22 ± 119.98^*∗*^	0.03	149.20 ± 8.90^*∗*^	<0.05
NC/24 h	1280.71 ± 174.95	—	659.93 ± 76.47	—	87.54 ± 3.37	—
Mann/24 h	1267.80 ± 141.28	1.00	593.29 ± 100.33	1.00	94.36 ± 12.63	1.00
H15/24 h	1899.81 ± 192.74^†^	0.02	1242.78 ± 157.80^†^	<0.05	137.62 ± 8.23^†^	<0.05
H25/24 h	2002.32 ± 227.84^†^	<0.05	1499.52 ± 178.26^†^	<0.05	205.16 ± 9.35^†^	<0.05
H50/24 h	1461.57 ± 204.17	0.99	1281.24 ± 185.36^†^	<0.05	167.17 ± 14.56^†^	<0.05
NC/48 h	1190.83 ± 88.05	—	604.00 ± 93.41	—	89.97 ± 9.13	—
Mann/48 h	1207.14 ± 244.60	1.00	590.15 ± 75.43	1.00	81.73 ± 9.56	1.00
H15/48 h	1351.34 ± 208.61	0.99	1145.16 ± 164.25^‡^	<0.05	140.06 ± 13.63^‡^	<0.05
H25/48 h	1813.77 ± 216.90^‡^	0.02	1286.16 ± 180.00^‡^	<0.05	186.33 ± 14.73^‡^	<0.05
H50/48 h	1301.38 ± 176.79	1.00	1047.77 ± 52.37^‡^	0.04	159.40 ± 14.21^‡^	<0.05

*N* = 3, ^*∗*^*P* < 0.05 compared with the NC group at 12 h; ^†^*P* < 0.05 compared with the NC group at 24 h, ^‡^*P* < 0.05 compared with the NC group at 48 h.

**Table 2:** The protein levels of TGF-*β*_1_, FN, and collagen I in different concentrations of thapsigargin at different times.

	TGF-*β*1 (ng/L)		FN (*μ*g/ml)		Coll I (ng/L)	
	Mean ± SD	*P*	Mean ± SD	*P*	Mean ± SD	*P*
NC	1214.17 ± 107.99	—	595.82 ± 96.72	—	87.53 ± 9.22	—
DMSO	1207.90 ± 77.80	1.00	607.90 ± 88.25	1.00	89.16 ± 12.42	1.00
Thap, 0.1/6 h	1324.93 ± 117.29	0.98	689.67 ± 98.39	0.95	117.98 ± 26.66	0.73
Thap, 0.1/24 h	1868.72 ± 121.23^*∗*^	<0.05	983.82 ± 100.21^*∗*^	<0.05	190.99 ± 25.37^*∗*^	<0.05
Thap, 0.1/48 h	1546.91 ± 150.47	0.20	755.08 ± 109.47	0.59	135.02 ± 24.07	0.51
Thap, 0.2/6 h	1318.07 ± 181.84	0.99	772.14 ± 113.70	0.47	138.21 ± 23.80	0.19
Thap, 0.2/24 h	1794.28 ± 157.11^*∗*^	<0.05	896.43 ± 105.48^*∗*^	0.04	157.87 ± 20.64^*∗*^	0.03
Thap, 0.2/48 h	1423.61 ± 242.25	0.70	744.88 ± 119.11	0.66	115.34 ± 26.64	0.80

*N* = 3, ^*∗*^*P* < 0.05 compared with the NC group.

## Data Availability

The data analyzed during this study are included within the article.
